# Transarterial chemoembolization combined with Jie-du granule preparation improves the survival outcomes of patients with unresectable hepatocellular carcinoma

**DOI:** 10.18632/oncotarget.16804

**Published:** 2017-04-03

**Authors:** Hetong Zhao, Xiaofeng Zhai, Zhe Chen, Xuying Wan, Lanyu Chen, Feng Shen, Changquan Ling

**Affiliations:** ^1^ Department of Traditional Chinese Medicine, The Changhai Hospital, Second Military Medical University, Shanghai, China; ^2^ Department of Combined Traditional Chinese and Western Medicine, The Eastern Hepatobiliary Surgery Hospital, Second Military Medical University, Shanghai, China; ^3^ Department of Hepatic Surgery, The Eastern Hepatobiliary Surgery Hospital, Second Military Medical University, Shanghai, China

**Keywords:** hepatocellular carcinoma, Jie-du granule, transarterial chemoembolization, retrospective cohort study

## Abstract

The aim of the present study was to compare the effectiveness of transarterial chemoembolization (TACE), TACE combined with Jie-du granules (JD), and TACE combined with sorafenib (SOR) for treating patients with unresectable hepatocellular carcinoma (HCC). For this purpose, we conducted a retrospective analysis of data from 266 consecutive patients with unresectable HCC who underwent TACE treatment at the Shanghai Hospital and Eastern Hepatic Surgery Hospital between Jan 2009 and Dec 2010. We prospectively analyzed patient survival and progression times as well as independent predictors, within a follow-up period of 86 months. Patients were divided into TACE-JD (*n* = 75), TACE-SOR (*n* = 124) and TACE (*n* = 67) groups. Median overall survival (OS) times being: TACE-JD, 21.43 months; TACE-SOR, 23.23 months; TACE, 13.97 months (TACE-SOR vs TACE, *P* < 0.001; TACE-SOR vs TACE-JD, *P* = 0.852; TACE-JD vs TACE, *P* < 0.001). The median times to progression (TTP) were as follows: TACE-JD, 8.67 months; TACE-SOR, 5.37 months; TACE, 4.57 months (TACE-SOR vs TACE, *P* = 0.479; TACE-SOR vs TACE-JD, *P* < 0.001; TACE-JD vs TACE, *P* < 0.001). Independent predictors of OS were treatment allocation, Child-Pugh class large tumor, albumin and extrahepatic metastasis. These findings show that patients with unresectable HCC who were administered TACE-JD survived significantly longer compared with those administered TACE or TACE-SOR.

## INTRODUCTION

Hepatocellular carcinoma (HCC) is the sixth most common cancer and second leading cause of cancer-related death worldwide [1–2]. Liver resection and transplantation may offer curative treatment for patients with HCC, and percutaneous radiofrequency ablation may have a potentially curative effect, but most patients are unable to undergo these treatments because of advanced disease upon diagnosis. Transarterial chemoembolization (TACE) is an effective, commonly used treatment for patients whose conditions are unsuitable for surgical or ablative procedures [3–4]. TACE combined with other treatments may improve the outcomes of patients with unresectable HCC. For example, bevacizumab [5], sorafenib [6], and arsenic trioxide [7] combined with TACE can prolong the survival of patients with HCC. Further, a meta-analysis found that TACE plus sorafenib (TACE-SOR) administered to patients with intermediate or advanced HCC improved overall survival (OS), time to progression (TTP), and the overall response rate [8–12]. However, a phase III randomized controlled trial found that that TACE-SOR did not clinically improve TTP compared with TACE in patients with intermediate stage multinodular HCC and that the time to unTACEable progression (TTUP) was lower for patients administered sorafenib compared with those given placebo [8].

The Chinese herbal formula Jie-du granule preparation (JD), which is widely used for treating HCC, comprises anticancer and detoxifying endotoxic Chinese herbal medicines prepared according to the theory of traditional Chinese herbal medicine. For example, a few studies have demonstrated that TACE combined with JD granules may prolong the survival of patients with unresectable HCC [13–16]. In the present study, we compared the efficacy of TACE, TACE plus JD (TACE-JD), and TACE-SOR for treating patients with unresectable HCC.

## RESULTS

### Patient characteristics

The baseline characteristics of the 266 patients among the three groups were not significantly different (Table 1). We then compared TTP and OS among the TACE-JD, TACE-SOR, and TACE groups to identify associated factors and establish the efficacy of TACE-JD compared with TACE-SOR and TACE alone.

**Table 1:** Patients’ baseline demographics, disease characteristics, and treatments

Treatment	TACE plus JD (*N* = 75)	TACE plus Sorafenib (*N* = 124)	TACE (*N* = 67)	*p* value
Age (yr)	55.2 ± 11.6	50.7 ± 12.2	51.7 ± 11.1	0.053
Gender				0.509
Male	66 (88.0%)	108 (87.1%)	62 (92.5%)	
Female	9 (12.0%)	16 (12.9%)	5 (7.5%)	
Child-pugh class				0.645
A	67 (89.3%)	107 (86.3%)	61 (91.0%)	
B	8 (10.7%)	17 (13.7%)	6 (9.0%)	
ECOG performance status				0.068
0	44 (58.7%)	77 (62.1%)	43 (64.2%)	
1	24 (32.0%)	36 (29.0%)	24 (35.8%)	
2	7 (9.3%)	11 (8.9%)	0 (0.0%)	
Number of tumors ^†^				< 0.001
Single	17 (23.3%)	61 (49.6%)	24 (35.8%)	
Multiple	50 (68.5%)	61 (49.6%)	43 (64.2%)	
Largest tumor size (cm)				0.003
< 5	33 (44.0%)	85 (68.5%)	38 (56.7%)	
≥ 5	42 (56.0%)	39 (31.5%)	29 (43.3%)	
HBSAG				0.159
Negative	19 (25.3%)	21 (16.9%)	9 (13.4%)	
Positive	56 (74.7%)	103 (83.1%)	58 (86.6%)	
Ascites				< 0.001
Absent	61 (81.3%)	119 (96.0%)	66 (98.5%)	
Present	14 (18.7%)	5 (4.0%)	1 (1.5%)	
Portal vein thrombosis				0.031
Absent	54 (73.0%)	89 (71.8%)	59 (88.1%)	
Present	20 (27.0%)	35 (28.2%)	8 (11.9%)	
Extrahepatic metastasis				0.727
Absent	58 (78.4%)	101 (81.5%)	56 (83.6%)	
Present	16 (21.6%)	23 (18.5%)	11 (16.4%)	
Cirrhosis				< 0.001
Absent	1 (1.3%)	68 (54.8%)	39 (58.2%)	
Present	74 (98.7%)	56 (45.2%)	28 (41.8%)	
Total bilirubin level (mmol/L)				0.207
< 18	54 (72.0%)	74 (59.7%)	44 (65.7%)	
≥ 18	21 (28.0%)	50 (40.3%)	23 (34.3%)	
Aspartate transaminase (IU/L)				0.115
< 64	52 (70.3%)	90 (72.6%)	39 (58.2%)	
≥ 64	22 (29.7%)	34 (27.4%)	28 (41.8%)	
Serum albumin (g/L)				0.022
< 35	53 (70.7%)	103 (83.1%)	59 (88.1%)	
≥ 35	22 (29.3%)	21 (16.9%)	8 (11.9%)	
a-Fetoprotein level (ng/mL)				0.043
< 500	53 (76.8%)	73 (58.9%)	44 (65.7%)	
≥ 500	16 (23.2%)	51 (41.1%)	23 (34.3%)	
Prothrombin Time (s)				0.01
< 14.3	48 (68.6%)	103 (83.1%)	59 (88.1%)	
≥ 14.3	22 (31.4%)	21 (16.9%)	8 (11.9%)	

Abbrevations: TACE, transarterial chemoembolization; JD, Jie-du; ECOG, Eastern Cooperative Oncology Group.

^†^Eight patients in the TACE plus JD group had missing data.

### OS and TTP

**Figure 1:**
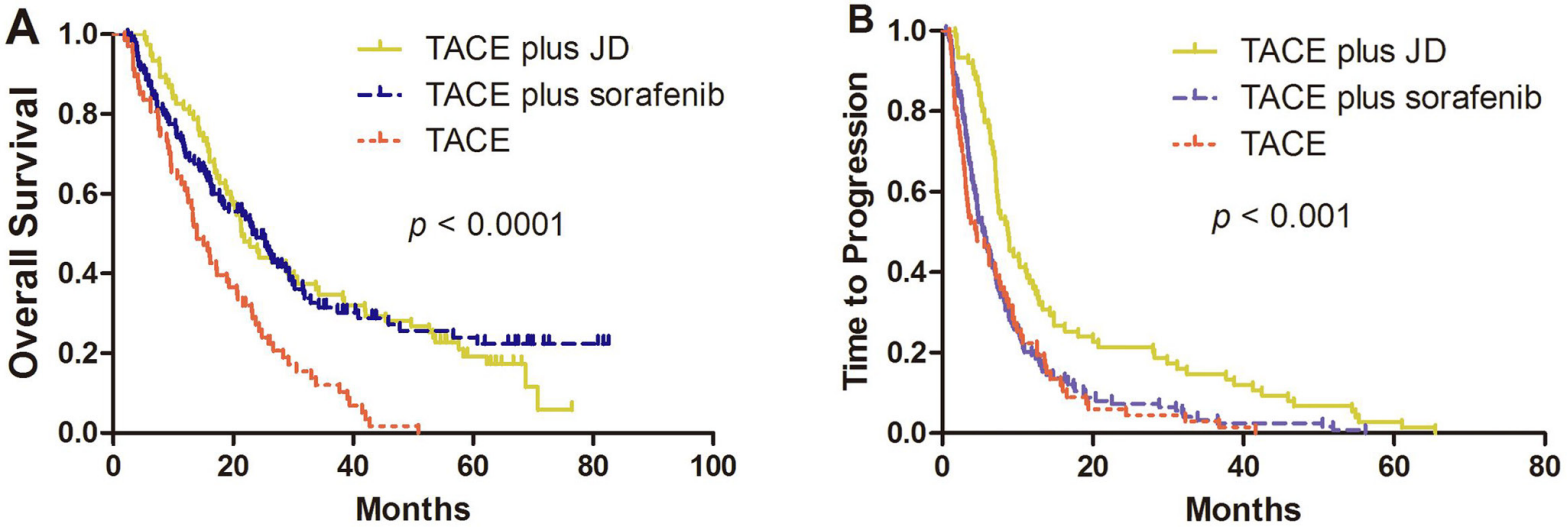
Kaplan–Meier curves of (**A**) OS and (**B**) TTP of patients with unresectable HCC who were administered TACE, TACE-SOR, or TACE–JD. (A) OS was significantly improved in the TACE-JD and TACE-SOR groups compared with TACE alone. TACE-JD vs TACE, *P* < 0.001; TACE-SOR vs TACE, *P* < 0.001; TACE-JD vs TACE-SOR, *P* = 0.852. (B) TTP was significantly improved by administration of TACE-JD compared with both TACE-SOR and TACE alone. TACE-JD vs TACE, *P* < 0.001; TACE-JD vs TACE-SOR, *P* < 0.001; TACE-SOR vs TACE, *P* = 0.476.

During the median follow-up of 18.76 months (range, 1–88 months), 210 patients (78.9%) died from HCC. The TACE-JD and TACE-SOR groups experienced significantly longer OS (21.43 months and 23.23 months, respectively) compared with that of the TACE group (13.97 months; *P* < 0.001) but there was no significant difference between the TACE-SOR and TACE-JD groups (*P* = 0.852) (Figure 1A). Additionally, the TACE-JD group experienced significantly longer TTP (8.67 months) compared with patients receiving TACE alone (4.57 months; *P* < 0.001) or TACE-SOR (5.37 months; *P* < 0.001). Furthermore, there was no significant difference between TACE-SOR and TACE only groups (*P* = 0.479) (Figure 1B).

### Independent prognostic factors associated with OS and TTP

Univariate analysis revealed that improved OS was significantly associated with treatment allocation, ECOG performance, large tumor size, extrahepatic metastasis, and aspartate transaminase (AST) (Table 2). Multivariate analysis identified treatment allocation, Child-Pugh class, large tumor size, albumin, and extrahepatic metastasis as independent predictors of OS (Table 3). Univariate analysis revealed that improved TTP was significantly associated with treatment allocation, age, portal vein tumor thrombosis, extrahepatic metastasis, and AST (Table 2). Multivariate analysis identified treatment allocation, AST, albumin, and extrahepatic metastasis as independent predictors of TTP (Table 4).

**Table 2:** Univariate analysis of prognostic factors for OS and TTP

Variable		*n* (%)	OS	TTP
			HR(95% CI), *p* value	HR(95% CI), *p* value
Treatment	TACE	67 (25.2%)	1 [Reference]	1 [Reference]
	TACE plus JD	75 (28.2%)	0.5 (0.3, 0.7) < 0.001	0.5 (0.4, 0.7) < 0.001
	TACE plus Sorafenib	124 (46.6%)	0.5 (0.3, 0.7) < 0.001	0.9 (0.7, 1.2) 0.557
Age	≤ 50	121 (45.5%)	1 [Reference]	1 [Reference]
	> 50	145 (54.5%)	0.9 (0.7, 1.2) 0.518	0.7 (0.6, 0.9) 0.007
Gender	Male	236 (88.7%)	1 [Reference]	1 [Reference]
	Female	30 (11.3%)	0.9 (0.6, 1.4) 0.665	0.8 (0.6, 1.2) 0.358
Child-pugh class	A	235 (88.3%)	1 [Reference]	1 [Reference]
	B	31 (11.7%)	1.0 (0.6, 1.5) 0.882	1.3 (0.9, 1.9) 0.168
ECOG performance status	0	164 (61.7%)	1 [Reference]	1 [Reference]
	1	84 (31.6%)	1.4 (1.1, 1.9) 0.022	1.3 (1.0, 1.6) 0.081
	2	18 (6.8%)	1.6 (1.0, 2.8) 0.075	1.2 (0.8, 2.0) 0.372
Number of tumors	Single	102 (39.8%)	1 [Reference]	1 [Reference]
	Multiple	154 (60.2%)	1.2 (0.9, 1.6) 0.268	1.1 (0.8, 1.4) 0.569
Largest tumor size (cm)	< 5	156 (58.6%)	1 [Reference]	1 [Reference]
	≥ 5	110 (41.4%)	1.7 (1.3, 2.2) < 0.001	1.2 (0.9, 1.5) 0.193
HBsAg	Absent	49 (18.4%)	1 [Reference]	1 [Reference]
	Present	217 (81.6%)	1.0 (0.7, 1.4) 0.950	1.0 (0.7, 1.4) 0.965
Ascites	Absent	246 (92.5%)	1 [Reference]	1 [Reference]
	Present	20 (7.5%)	0.9 (0.5, 1.5) 0.695	0.9 (0.6, 1.4) 0.684
Portal vein thrombus	Absent	202 (76.2%)	1 [Reference]	1 [Reference]
	Present	63 (23.8%)	1.3 (1.0, 1.8) 0.098	1.4 (1.0, 1.8) 0.029
Extrahepatic metastasis	Absent	215 (81.1%)	1 [Reference]	1 [Reference]
	Present	50 (18.9%)	1.7 (1.2, 2.4) 0.002	1.8 (1.3, 2.5) < 0.001
Cirrhosis	Absent	108 (40.6%)	1 [Reference]	1 [Reference]
	Present	158 (59.4%)	0.9 (0.7, 1.2) 0.422	0.7 (0.5, 0.9) 0.003
Total bilirubin level (mmol/L)	< 18	172 (64.7%)	1 [Reference]	1 [Reference]
	≥18	94 (35.3%)	1.2 (0.9, 1.6) 0.268	1.2 (0.9, 1.5) 0.181
Aspartate transaminase (IU/L)	< 64	181 (68.3%)	1 [Reference]	1 [Reference]
	≥ 64	84 (31.7%)	1.5 (1.1, 2.0) 0.005	1.5 (1.2, 2.0) 0.001
Serum albumin (g/L)	≥ 35	215 (80.8%)	1 [Reference]	1 [Reference]
	< 35	51 (19.2%)	1.1 (0.8, 1.5) 0.690	1.1 (0.8, 1.5) 0.509
a-Fetoprotein level (ng/mL)	< 400	170 (65.4%)	1 [Reference]	1 [Reference]
	≥ 400	90 (34.6%)	1.1 (0.8, 1.5) 0.407	1.1 (0.9, 1.5) 0.300
Prothrombin Time (s)	< 14.3	210 (80.5%)	1 [Reference]	1 [Reference]
	≥14.3	51 (19.5%)	0.8 (0.6, 1.2) 0.241	1.0 (0.7, 1.3) 0.935

Abbrevations: OS, overall survival; TTP, time to progression; HR, hazard ratio; TACE, transarterial chemoembolization; JD, Jie-du; ECOG, Eastern Cooperative Oncology Group.

**Table 3:** Multivariate analysis of prognostic factors for OS

Variable		*n* (%)	OS
			HR (95% CI), *p* value
Treatment	TACE	67 (25.2%)	1 [Reference]
	TACE plus JD	75 (28.2%)	0.4 (0.2, 0.6) < 0.001
	TACE plus Sorafenib	124 (46.6%)	0.5 (0.4, 0.8) 0.002
Child-pugh class	A	235 (88.3%)	1 [Reference]
	B	31 (11.7%)	0.5 (0.3, 1.0) 0.035
Largest tumor size (cm)	< 5	156 (58.6%)	1 [Reference]
	≥ 5	110 (41.4%)	1.8 (1.2, 2.5) 0.002
Extrahepatic metastasis	Absent	215 (81.1%)	1 [Reference]
	Present	50 (18.9%)	1.6 (1.1, 2.4) 0.026
Serum albumin (g/L)	≥ 35	215 (80.8%)	1 [Reference]
	< 35	51 (19.2%)	1.6 (1.1, 2.6) 0.029

Abbrevations: OS, overall survival; HR, hazard ratio; TACE, transarterial chemoembolization; JD, Jie-du.

**Table 4:** Multivariate analysis of prognostic factors for TTP

Variable		*n* (%)	TTP
			HR(95% CI), *p* value
Treatment	TACE	67 (25.2%)	1 [Reference]
	TACE plus JD	75 (28.2%)	0.5 (0.4, 0.8) 0.005
	TACE plus Sorafenib	124 (46.6%)	1.0 (0.7, 1.4) 0.978
Extrahepatic metastasis	Absent	215 (81.1%)	1 [Reference]
	Present	50 (18.9%)	1.8 (1.2, 2.6) 0.004
Aspartate transaminase (IU/L)	< 64	181 (68.3%)	1 [Reference]
	≥ 64	84 (31.7%)	1.6 (1.2, 2.2) 0.004
Serum albumin (g/L)	≥ 35	215 (80.8%)	1 [Reference]
	< 35	51 (19.2%)	1.6 (1.1, 2.3) 0.027

Abbrevations: TTP, time to progression; TACE, transarterial chemoembolization; JD, Jie-du.

### Comparison of cost-effectiveness

Although there were no significant differences in hospitalization costs between the three treatment groups, admission costs of the TACE-JD group were significantly lower compared with those of the TACE-SOR group (*P* < 0.001) (Table 5).

**Table 5:** Total hospitalization and admissions costs

Cost (month)	TACE+JD (*N* = 75)	TACE+Sorafenib (*N* = 124)	TACE (*N* = 67)	*p* value
Hospitalization costs (dollar)	1570.60 ± 748.53	1997.94 ± 927.39	1830.30 ± 612.83	0.057
Admission costs (dollar)	117.47 ± 17.61	7333.81	0	< 0.001

Abbrevations: TACE, transarterial chemoembolization; JD, Jie-du.

## DISCUSSION

Herein we compared OS and TTP of patients with unresectable HCC who were treated with TACE alone, TACE-SOR or TACE-JD. We found that the combination therapies, TACE-JD and TACE-SOR, were associated with improved OS compared with TACE alone, and TACE-JD was associated with longer TTP compared with both TACE-SOR and TACE alone.

The development and progression of HCC is complex. First-line treatment for intermediate and advanced HCC in China includes TACE or sorafenib monotherapy [19–21]. The ranges of OS achieved using TACE and TACE-SOR to treat patients with unresectable HCC are 5.1–17 months and 7.5–27 months, respectively [22–23], which is consistent with the results of the present study. The results of the phase II SPACE trial show that TACE-SOR does not improve TTP compared with TACE plus placebo in Western or Eastern patients, [8, 24] which is also in agreement with our findings.

Oncologists state that traditional Chinese medicine should be involved in all aspects of HCC treatment [25–26]. For example, a retrospective study showed that TACE combined with a JD granule preparation may prolong survival of patients with unresectable HCC (9.2 vs. 5.87 months, *P* < 0.01) [13]. The composition and dose of the JD preparation were equivalent to that of the JD used in the present study. Although OS was similar between the TACE-SOR and TACE-JD groups, secondary and exploratory analyses of TTP suggest that JD may have slowed tumor growth and metastasis. Given the disease complexity characterized by the interplay between HCC and underlying liver disease, TTP serves as a surrogate for OS [27–28]. These results highlight the requirement for prospective studies to define relevant surrogate endpoints for OS.

Prognoses for HCC differ because of the influence of numerous factors [29]. After adjusting for significant factors revealed by univariate analysis, multivariate analysis identified treatment allocation, large tumor size and extrahepatic metastasis as independent predictors of OS and that treatment allocation, Barcelona Clinic Liver Cancer stage and AST were independent predictors of TTP. These results are consistent with the findings of previous studies [29–30]. For example, AST is a significant predictor of survival after diagnosis of HCC in patients with chronic HBV infections, which is in agreement with our findings that AST was an independent predictor of TTP [30].

Our data show that TACE-JD was more economical compared with TACE-SOR. Cost-effectiveness was evaluated roughly by comparing total hospital and admission costs. There were no significant differences between the two combination treatment groups in terms of total hospital costs; however, the cost of admission was lower for the TACE-JD group compared with the TACE-SOR group, which may be explained by the low price of Chinese herbs compared with the high cost of sorafenib.

The present study has limitations such as the relatively small number of nonrandomized patients, its retrospective nature, and the different treatment periods. However, the safety and effectiveness of the treatments were consistent with those of previous randomized trials. In conclusion, our study suggests that combined treatment with TACE and JD is feasible and beneficial for patients with unresectable HCC. Moreover, TACE-JD was more cost-effective in terms of admission expenses. Further studies are therefore warranted to confirm the efficacy of TACE combined with JD for patients with unresectable HCC.

## MATERIALS AND METHODS

### Patients

In this retrospective study, we collected data for 266 patients with unresectable HCC treated with TACE between January 2009 and December 2010 at the Shanghai Hospital and Eastern Hepatic Surgery Hospital. Patients were followed until April 2016. Patients were allocated into three groups according to the type of initially proposed therapy. Inclusion criteria were as follows: i) HCC diagnosed according to noninvasive criteria consistent with the guidelines of the European Association for the Study of Liver/American Association for the study of Liver Disease or pathological diagnosis; ii) Child-Pugh class A or B liver function; iii) Eastern Cooperative Oncology Group (ECOG) performance score ≤ 2; iv) HCC deemed to be unresectable upon review of cross-sectional images of the tumors by two experienced liver surgery specialists at the participating centers; v) Availability of complete medical records and prognostic data. Contrast-enhanced computed tomography (CT) and magnetic resonance imaging (MRI) were used to detect and diagnose infiltrating HCC. Written informed consent was obtained from all patients at initiation of treatment. Approval of the retrospective analysis by the institutional ethics committee was not required. The study protocol followed appropriate guidelines of the Declaration of Helsinki.

### Treatment methods

TACE was performed by delivering selective transarterial chemotherapy into the vessels feeding the tumor using an emulsion of lipiodol (5–20 ml) and doxorubicin (30–60 mg) followed by embolization with absorbable particles (gel foam). The TACE-SOR group was initially administered sorafenib (400 mg) orally twice each day, 3–5 days after the first TACE session, and patients received continuous sorafenib before or after repeated TACE. The dose of sorafenib was reduced according to the presence of toxicity. If grade 3 or 4 hematological, skin or gastrointestinal toxicity, hypertension, or hepatic dysfunction (defined by the National Cancer Institute Common Terminology Criteria for Adverse Events version 3.0) [17] occurred, the dose was adjusted to 400 mg once daily until the adverse events were alleviated or eliminated. If grade 3 or 4 adverse events continued after dose adjustment, sorafenib treatment was halted until the adverse effects were alleviated or disappeared.

The JD preparation comprised the traditional Chinese herbal medicines as follows: root of *Actinidia valvata*, root of *Salvia chinensis*, bulb of *Cremastra appendiculata*, and gizzard membrane of *Gallus gallus domesticus* (1:1:0.4:0.4), which were extracted using hot water and then lyophilized. In the TACE-JD group, 6 g (equivalent to 80 g of raw herbal material) of the JD preparation (Tianjiang Pharmaceutical Factory, Jiangsu, China; Production License No. Su ZzY20010266) was administered twice each day, 30 min after meals.

### Follow-up

Patients were examined as outpatients once every three months after treatment using clinical examination, biochemical analysis, and measurements of serum α-fetoprotein. The response of tumors to TACE was evaluated using contrast-enhanced CT or MRI once every 2–3 months after treatment. The modified Response Evaluation Criteria in Solid Tumors (RECIST) definitions were applied to the CT or MRI data to measure tumor response [18], and patients’ Child-Pugh classes and ECOG scores were recorded.

### Endpoints

Tumor response to TACE was evaluated 3 months after treatment using contrast-enhanced CT or MRI. The presence of unenhanced tumor areas reflected tissue necrosis. The modified RECIST definitions were applied to CT or MRI data to measure tumor response [18].

### Statistical analysis

Statistical analyses were performed using SPSS 19.0 software (SPSS, Inc., Chicago, IL). Comparisons between two groups were performed using the Student *t* test for continuous data and the chi-square test for categorical data. OS was calculated using a life-table method and compared with the results of the Mantel-Cox test. Survival curves were generated using the Kaplan–Meier method and compared using the log-rank test. The relative prognostic significance of the variables for predicting OS rates was assessed using multivariate Cox proportional hazards regression analysis. The results are presented as the means ± standard deviation or median and range. All statistical tests were two-sided and *P* < 0.05 indicates a significant difference.
